# 51. Patient Reported Outcomes Collection: A Mixed Methods Study at an urban HIV Clinic associated with a Historically Black Medical College in the Southern United States

**DOI:** 10.1093/ofid/ofab466.051

**Published:** 2021-12-04

**Authors:** Paul Parisot, Facerlyn Wheeler, Kemberlee Bonnet, Peter F Rebeiro, Korlu McCainster, Robert Cooper, Vladimir Berthaud, David Schlundt, April Pettit

**Affiliations:** 1 Vanderbilt University Medical Center, Nashville, Tennessee; 2 Meharry Medical College, Nashville, Tennessee; 3 Vanderbilt University, Nashville, TN; 4 MEHARRY MEDICAL COLLEGE, NASHVILLE, Tennessee

## Abstract

**Background:**

Black Americans, particularly in the South, are disproportionately affected by the US HIV epidemic. We piloted the use of an electronic tablet to collect patient reported outcomes (PRO) data on social and behavioral determinants of health among people with HIV (PWH) at the Meharry Community Wellness Center (MCWC), an HIV clinic affiliated with a Historically Black Medical College in Nashville, Tennessee. Studies have shown PRO collection can improve patient outcomes and provide oft-overlooked data on mental health, substance use, and patient adherence to ART.

**Methods:**

We enrolled 100 PWH in care at the MCWC consecutively to complete validated PRO tools (Table 1) using the Research Electronic Data Capture (REDCap) platform on a hand-held tablet. Using a purposive sampling strategy, we enrolled 20 of the 100 participants in an in-depth interview (IDI). Interview guide development was grounded in the Cognitive Behavioral Model in which thoughts, feelings, and behaviors are inter-related. IDIs were audio recorded, transcribed, de-identified, and formatted for coding. A hierarchical coding system was developed and refined using an inductive-deductive approach.

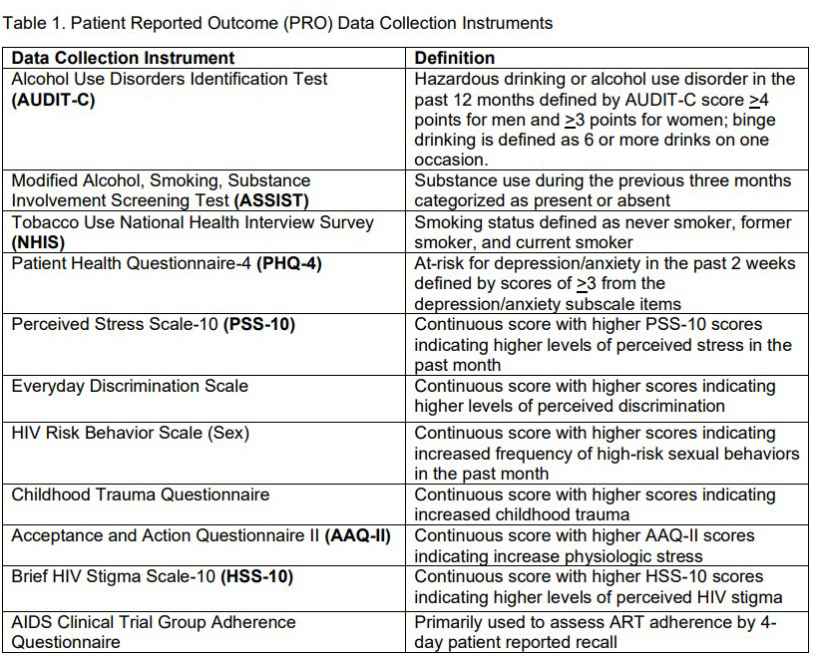

**Results:**

Among 100 PWH enrolled, median age was 50 years, 89% were Black, 60% were male, and 82% were living below 100% of the Federal Poverty Level. IDI participants felt the tablet was easy to use and the question content was meaningful. Question content related to trauma, sexual and drug use behaviors, mental health, stigma, and discrimination elicited uncomfortable or distressing feelings in some participants. Patients expressed a strong desire to be truthful and most would complete these surveys without compensation at future visits if offered.

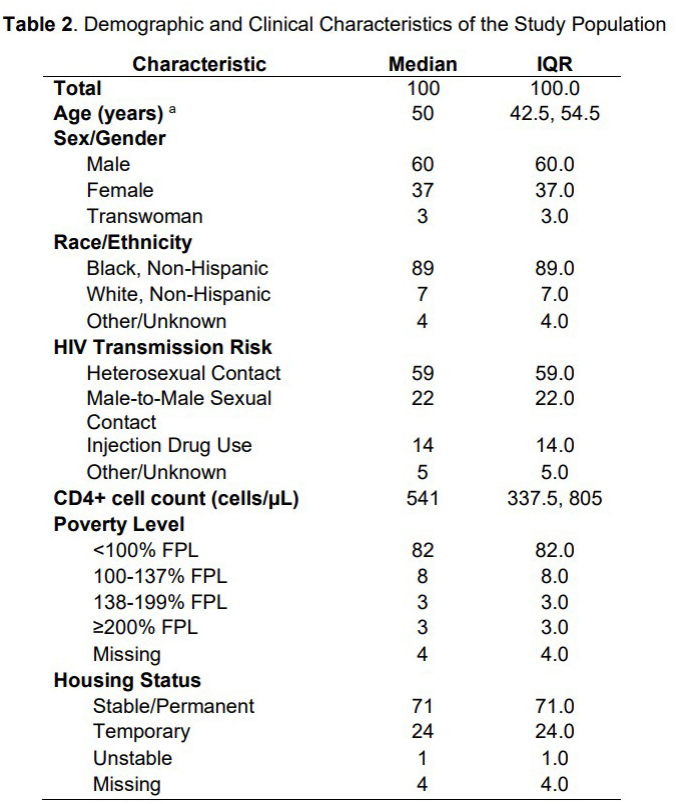

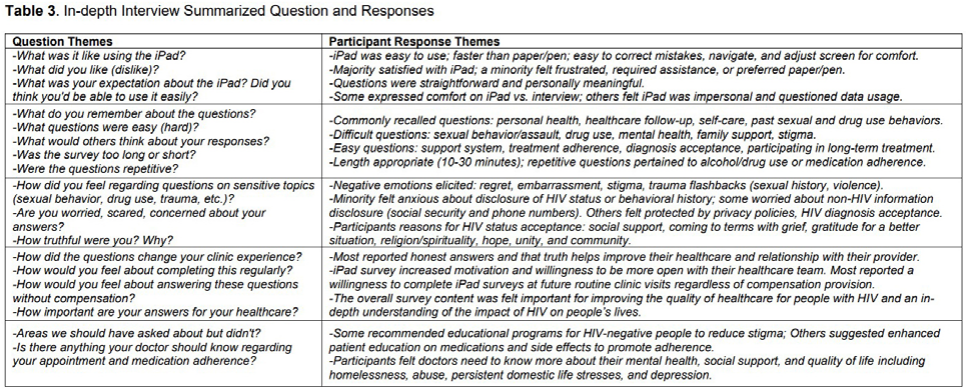

**Conclusion:**

The use of an electronic tablet to complete PRO data collection was feasible and well received by this cohort of vulnerable persons in HIV care in the US South. Despite some discomfort, our cohort overwhelmingly believed this was a valuable part of their medical experience. Real-time PRO data collection allows providers to screen for and act on social and behavioral determinants of health. Future research will focus on scaling up the implementation and evaluation of PRO data collection in a contextually appropriate manner.

**Disclosures:**

**Peter F. Rebeiro, PhD, MHS**, **Gilead** (Other Financial or Material Support, Single Honorarium for an Expert Panel)

